# Enrichment of risk SNPs in regulatory regions implicate diverse tissues in Parkinson’s disease etiology

**DOI:** 10.1038/srep30509

**Published:** 2016-07-27

**Authors:** Simon G. Coetzee, Steven Pierce, Patrik Brundin, Lena Brundin, Dennis J. Hazelett, Gerhard A. Coetzee

**Affiliations:** 1 Bioinformatics and Computational Biology Research Center, Biomedical Sciences, Cedars-Sinai Medical Center, Los Angeles, California, USA; 2 Center for Neurodegenerative Science, Van Andel Research Institute, Grand Rapids, Michigan, USA

## Abstract

Recent genome-wide association studies (GWAS) of Parkinson’s disease (PD) revealed at least 26 risk loci, with associated single nucleotide polymorphisms (SNPs) located in non-coding DNA having unknown functions in risk. In order to explore in which cell types these SNPs (and their correlated surrogates at r^2^ ≥ 0.8) could alter cellular function, we assessed their location overlap with histone modification regions that indicate transcription regulation in 77 diverse cell types. We found statistically significant enrichment of risk SNPs at 12 loci in active enhancers or promoters. We investigated 4 risk loci in depth that were most significantly enriched (−log_e_P > 14) and contained 8 putative enhancers in the different cell types. These enriched loci, along with eQTL associations, were unexpectedly present in non-neuronal cell types. These included lymphocytes, mesendoderm, liver- and fat-cells, indicating that cell types outside the brain are involved in the genetic predisposition to PD. Annotating regulatory risk regions within specific cell types may unravel new putative risk mechanisms and molecular pathways that contribute to PD development.

Parkinson’s disease (PD) is the second most common neurodegenerative disorder, affecting about 1% of those over the age of 60. Classically, PD was considered a movement disorder with akinesia, rigidity, tremor and postural instability as the predominant motor features. More recently, it became clear that patients also experience a wide range of disabling nonmotor disturbances, e.g. olfactory deficits, sleep disorders, depression, cognitive decline, constipation and other autonomic changes^1^. The neuropathology is characterized by severe degeneration of substantia nigra dopaminergic neurons (crucial for the motor deficits) and progressive development of intraneuronal α-synuclein aggregates throughout the central and peripheral nervous systems^2^. Monogenetic forms of PD are rare and only account for 5–10% of cases^3^ (see also longer reviews^4^,^5^,^6^); a total of 7 autosomal and recessive genes have been identified, exhibiting varying degrees of penetrance. Thus, the vast majority of cases of PD are sporadic and a combination of environmental factors and complex genetic loci likely play a causative role^4^. In addition to the rare Mendelian-inherited cases of PD, the genetic predisposition to PD includes at least 26 common single nucleotide polymorphism (SNP) variants (“index SNPs”), each imposing low but significant risk^7^,^8^. These risk loci associated with PD were discovered by population-based genome-wide association studies (GWAS), published in 2014 in a comprehensive large-scale meta-analysis of reproducible hits^7^ and conveniently summarized in a Cell snapshot^3^. Importantly, most (>90%) of the identified index SNPs in PD GWAS^7^,^8^ are located in noncoding DNA regions, making the assignment of potential functionality or causality-even the identification of the specific genes associated with the risk-SNPs-quite challenging.

Since 2005, over 1,600 significant SNP risk variants have been identified by GWAS for more than 250 traits, many with complex genetic predisposition and unknown gene involvements (https://www.genome.gov/26525384). Most genetic epidemiological studies have attempted to characterize the risk SNPs found in non-coding DNA simply by considering the “nearest gene” as the one being involved in risk. However, many index SNPs and their surrogates [in linkage disequilibrium (LD)] reside in regulatory regions (mainly in enhancers) and the target genes of such regulation are most likely not the nearest genes^9^. Therefore, there is still a significant gap between the identification of most risk SNPs and an understanding of their biological function in human disease. Here, we have addressed this conundrum via the development and use of two Bioconductor software tools, FunciSNP and motifbreakR^10^,^11^, to functionally annotate risk SNPs for PD. The approach allows the identification of risk SNP enrichment at active regulatory elements in non-coding DNA (promoters and enhancers, in this study). Enrichment is defined as the presence of risk SNPs nonrandomly distributed and at an increased prevalence within chromatin regions with putative functions. The two alleles of the SNP may each impose a differential risk by affecting the regulatory capacity of the region on target genes.

In the past, we and others have used bioinformatic tools, including FunciSNP and motifbreakR, to interrogate genetic studies in light of both public and in-house next generation sequencing data. Noncoding risk regions of many complex diseases have thus been investigated in recent years. However, in most cases, this was done only in one specific cell- or tissue type within databases. More recently, with the curation of epigenetic data within the Roadmap Epigenomics Mapping Consortium (REMC), the mapping of risk alleles in a variety of cell types^12^ became possible. Thus, our present approach is based on the assessment of PD risk SNP enrichment at specific chromatin regulatory regions in multiple distinct cell- and tissue types. Twenty-one PD risk loci were assessed in 77 REMC cell types of diverse tissue- and lineage-origin. Eight enhancers at the four most significantly enriched PD risk loci were annotated in detail. They were found respectively in lymphocytes, mesendoderm, brain-, liver- and fat cells, allowing refined hypotheses to be formulated for the genetic predisposition to PD at these loci.

## Results

**Figure 1:**
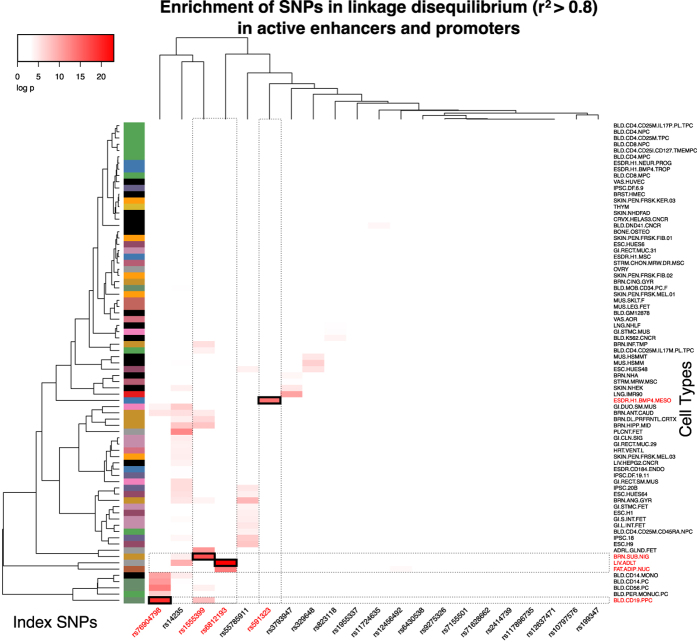
Tissue specific enrichment at PD GWAS loci. The relative enrichment of 21 index SNP (*x* axis) is shown by multiple hypothesis adjusted p-value (negative natural log) for each of the 77 REMC tissues (*y* axis). The data from Supplemental Table 1 are plotted; color from white to red indicates increasingly significant hits. SNPs and tissues selected for in-depth analysis are highlighted with red type on the axes and with guidelines on the plot. The specific SNP–tissue interactions that appear in subsequent figures are outlined in black.

We assessed 21 PD risk loci in 77 REMC tissues and cell types (supplemental Tables 1 and 2). One of the 77 REMC tissues was derived from human substantia nigra, which contains the dopaminergic neurons that undergo extensive degeneration in PD. The majority of the data in REMC are from tissues or cells that classically have not been considered to be affected by PD and have, so far, not been implicated in the disease development. We collected three data types. They were histone H3K4 mono and trimethylation (H3K4me1&3), and acetylation of H3K27 (H3K27ac). For the purposes of the present analyses, we restricted ourselves to active regulatory sequences in non-coding DNA (The data from Supplemental Table 1 are presented as a heat-map in Fig. 1). Regions with positive signals for the histone modification H3K4me3 were classified as promoters, and we considered them to be active if they overlapped with the active histone mark H3K27ac. On the other hand, we classified H3K4me1 regions as enhancers, and similarly we considered them to be active if there was overlap with H3K27ac. We compared enrichment of risk SNPs with all 1000 genomes SNPs within a window of 1 MB around each GWAS index SNP that we had found to occur within enhancer/promoter regions in the 77 REMC cell types. To this end, we counted correlated SNPs (at r^2^ ≥ 0.8; denoted as risk SNPs) at each risk locus in each of the 77 REMC cell types. We statistically assessed them (risk SNPs in regulatory regions vs. non-risk 1000genomes SNPs in regulatory regions) for significant differences, using the hypergeometric test after locus-specific corrections for multiple hypotheses (Supplemental Table 1 and Fig. 1). A schematic presentation of the workflow is found in Supplemental Fig. 1.

### Enrichment

**Figure 2:**
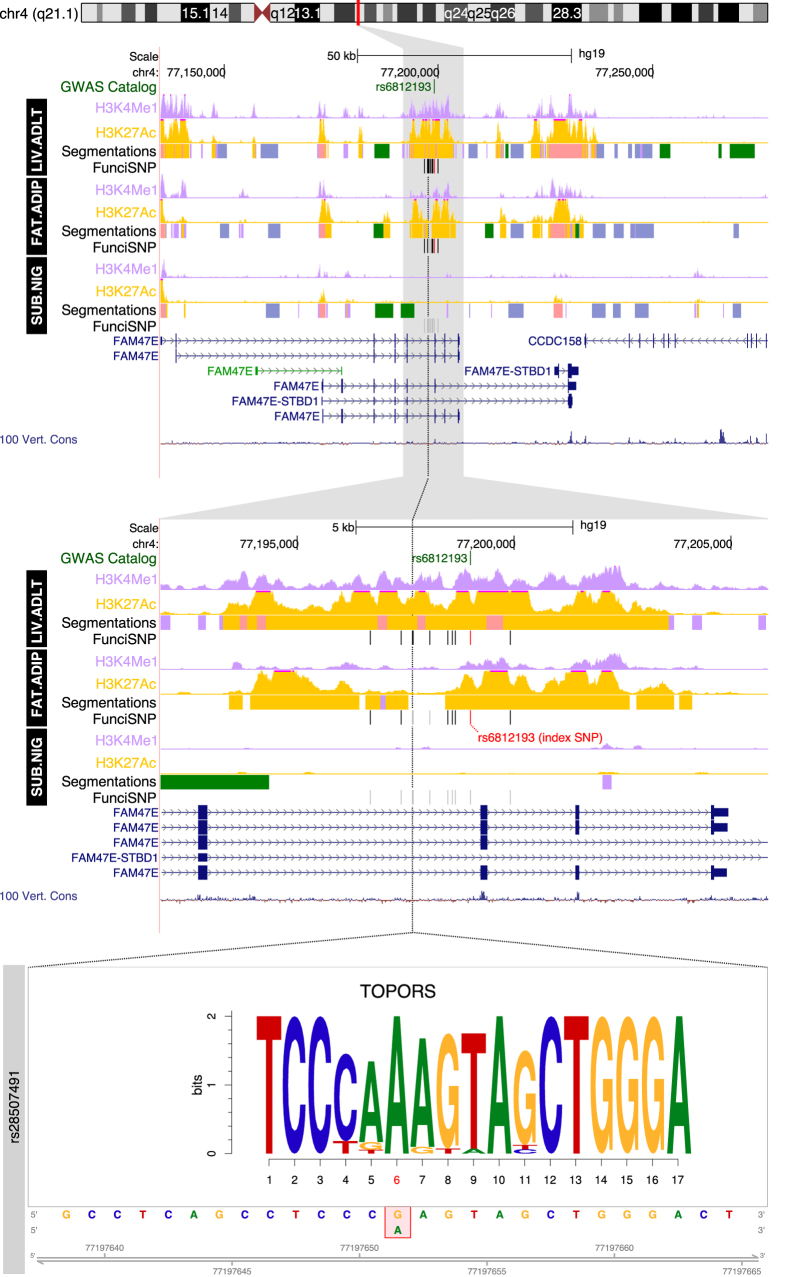
Functional enrichment at the 4q21 locus. Segmentation tracks summarize the functional annotations according to the REMC color scheme: orange for EAR (active enhancer), rose for PAR (active promoter), light purple for EPR and PPR (poised enhancers and promoters), grey for SCR (silenced regions), blue-grey for HET (heterochromatic) and green for TRS (transcribed). Wiggle tracks for H3K4me1 and H3K27Ac show the fine-grained epigenomics context in the regions surrounding each locus. The location of SNPs that disrupt transcription factor motifs are indicated with a dotted black line and an expanded view at the bottom. The motif is aligned with the genomic sequence surrounding the SNP, whose position is indicated in red; reference and alternate alleles are indicated in the red bounding box in the sequence below.

**Figure 3:**
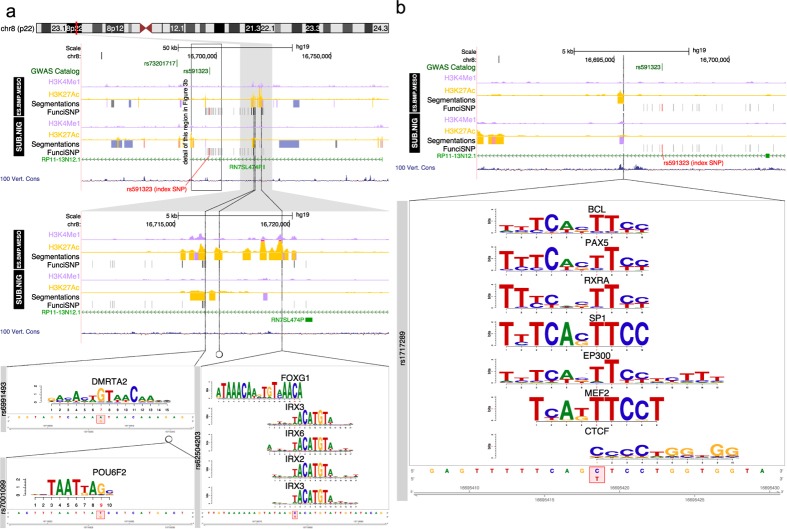
Functional enrichment at the 8p22 locus. (**a,b**) Two different regions at 8p22. Segmentation tracks summarize the functional annotations according to the REMC color scheme: orange for active enhancer, rose for active promoter, light purple for poised enhancers and promoters, grey for silenced regions, blue-grey for heterochromatin and green for transcribed regions. Wiggle tracks for H3K4me1 and H3K27ac show the fine-grained epigenomics context in the regions surrounding each locus. The location of SNPs that disrupt transcription factor motifs are indicated with a dotted black line and an expanded view at the bottom. The motif is aligned with the genomic sequence surrounding the SNP, whose position is indicated in red; reference and alternate alleles are indicated in the red bounding box in the sequence below.

**Figure 4:**
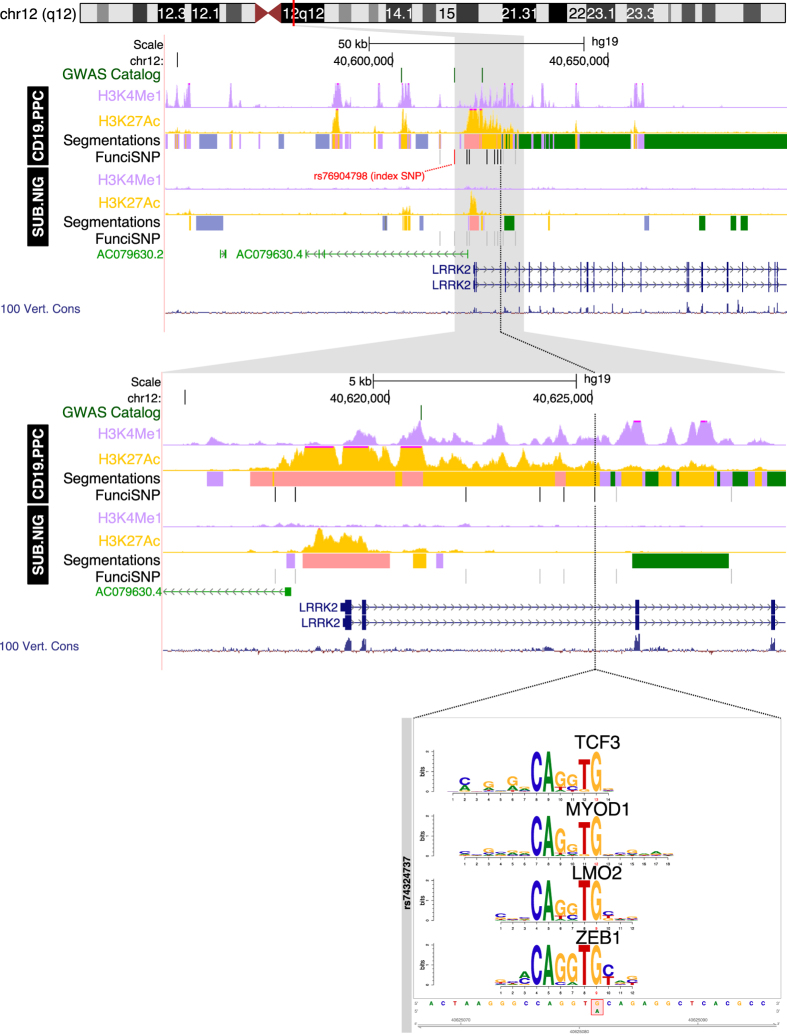
Functional enrichment at the 12q12 locus. Segmentation tracks summarize the functional annotations according to the REMC color scheme: orange for active enhancer, rose for active promoter, light purple for poised enhancers and promoters, grey for silenced regions, blue-grey for heterochromatin and green for transcribed regions. Wiggle tracks for H3K4me1 and H3K27ac show the fine-grained epigenomics context in the regions surrounding each locus. The location of SNPs that disrupt transcription factor motifs are indicated with a dotted black line and an expanded view at the bottom. The motif is aligned with the genomic sequence surrounding the SNP, whose position is indicated in red; reference and alternate alleles are indicated in the red bounding box in the sequence below.

**Figure 5:**
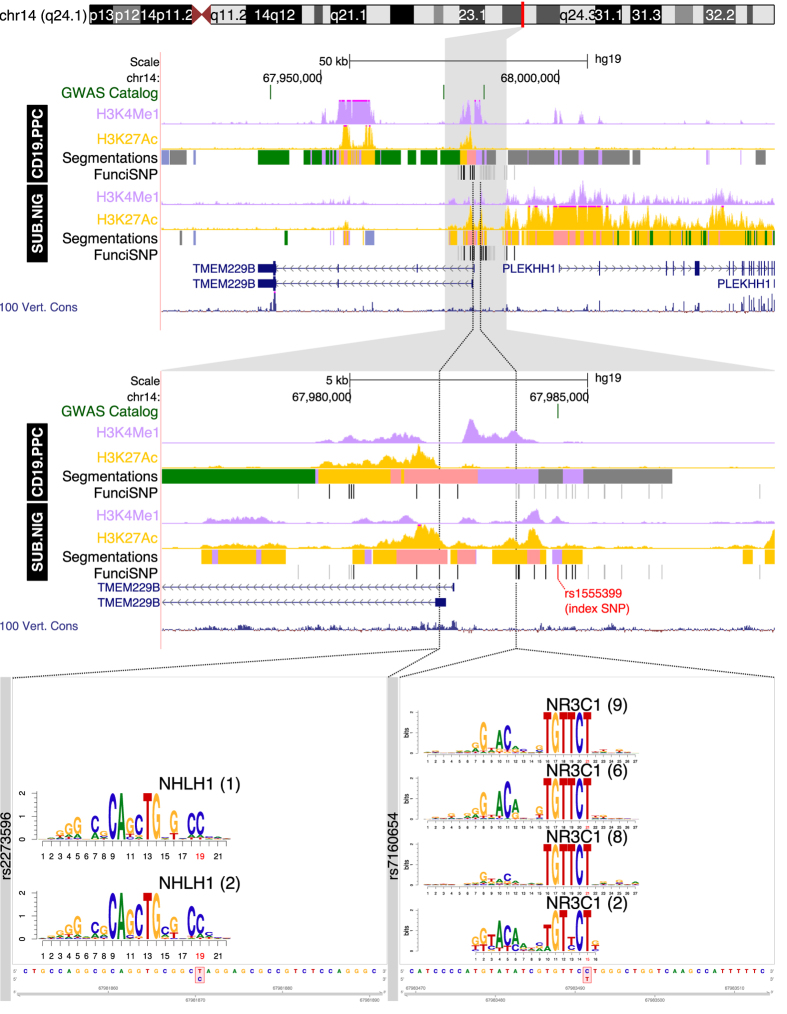
Functional enrichment at the 14q24 locus. Segmentation tracks summarize the functional annotations according to the REMC color scheme: orange for active enhancer, rose for active promoter, light purple for poised enhancers and promoters, grey for silenced regions, blue-grey for heterochromatin and green for transcribed regions. Wiggle tracks for H3K4me1 and H3K27ac show the fine-grained epigenomics context in the regions surrounding each locus. The location of SNPs that disrupt transcription factor motifs are indicated with a dotted black line and an expanded view at the bottom. The motif is aligned with the genomic sequence surrounding the SNP, whose position is indicated in red; reference and alternate alleles are indicated in the red bounding box in the sequence below.

We found enrichment of risk SNPs at 12 of the 21 loci, mainly at enhancers and not exclusively in nervous tissues, suggesting that the etiology of genetic risk is not restricted to the central nervous system. Based on very high significance (−log_e_P > 14) of enrichment, we chose to analyze eight putative enhancers in depth, as a proof-of-principle that our approach generates interesting and novel hypotheses about etiological and pathogenetic mechanisms of PD. The eight putative enhancers were at 4 most significantly enriched loci. They were at chromosome 4q21 (SNP enrichment in liver and fat cells, Fig. 2), chromosome 8p22 (SNP enrichment in BMP4 treated ES-derived mesendoderm, Fig. 3) and 12q12 (SNP enrichment in CD19-B lymphocytes, Fig. 4), and chromosome 14q24 (SNP enrichment in CD19-B lymphocytes and substantia nigra cells, Fig. 5).

### Locus 4q21.1

SNP enrichment was found at active enhancers in both liver and fat cells, but not in substantia nigra cells at 4q21.1 (Fig. 2). In both active cases the regulatory regions are likely to be super-enhancers^13^ (a.k.a. stretch enhancers^14^) each spanning some 10 kb. One of the enriched SNPs disrupts topoisomerase I binding, an arginine/serine-rich, E3 ubiquitin protein ligase (TOPORS). TOPORS is a nuclear protein and contains a RING-type zinc finger domain. It functions as an ubiquitin-protein E3 ligase^15^ but its function as a DNA binding protein is unknown. The response element (binding motif) falls within an intron for five isoforms of FAM47E. FAM47E is a protein of unknown function, but it is likely involved in inflammation and within epithelial biology networks^16^.

### Locus 8p22

Four putative active risk enhancers at chromosome 8p22 demonstrate risk-SNP response-element disruption in BMP4-treated ES-derived mesendoderm, but not in substantia nigra cells (Fig. 3A,B). Three of the enhancers (Fig. 3A) are either independent or form part of a super/stretch-enhancer^13^,^14^ spanning about 5 kb. Three SNPs (rs6991493, rs7001099, and rs62504203) disrupt response elements in DMRTA2, POU6F2, and FOXG1/IRX2,3&6, respectively. DMRTA2 (doublesex and mab-3 related transcription factor-like family A2) is mutated in novel cortical brain malformation^17^. POU6F2 (POU-domain homeobox transcription factor class 5) is associated with autism risk^18^. FOXG1 (forkhead box G1) is a transcription factor involved in the dysregulation of GABA/glutamate neuron differentiation in autism spectrum disorders^19^. IRX2,3&6 (Iroquois homeobox transcription factors) are involved in internal organ development such as the heart^20^. The fourth SNP (rs1717289) right next to H3K27ac mark in BMP4 treated ES-derived mesendoderm, disrupts several transcription response elements including a CTCF site (Fig. 3B). The CTCF site may demarcate enhancer/promoter loops important in distal enhancer regulation. The transcription factors in question RXRA, BCL, PAX5, SP11, EP300, and MEF2 are all involved in cell type differentiation in some way or another. All four enhancers are located in a large intron of the gene RP11-13N12.1, which encodes for a lincRNA of unknown function.

### Locus 12q12

A putative enhancer cluster at chromosome 12q12 is within an active enhancer region (H3K4me1 & H3K27ac) in CD19 B-lymphocytes cells but not brain cells. A strong promoter (H3K27ac) is also nearby. The enhancer is likely a super/stretch-enhancer^13^,^14^ spanning about 8 kb. A risk SNP (rs74324737) in the vicinity disrupts the response elements of four transcription factors with very similar response elements, namely TCF3, MYOD1, LMO2 and ZEB1 (Fig. 4). All four transcription factors are involved in tissue-specific signaling. The enhancer is within intron 2 of a multi-domain leucine-rich repeat kinase 2 (*LRRK2*) gene, which encodes a kinase regulating a subset of Rab GTPases and is known to give rise to autosomal dominant PD when mutated^21^.

### Locus 14q24.1

Two very strong risk enhancers (as judged by the type, extent and sizes of the modified histone peaks) likely forming part of another super/stretch-enhancer^13^,^14^ (spanning about 5 kb), are located at chromosome 14q24.1 (Fig. 5). They are found specifically in substantia nigra and to a lesser extent in CD19 B-lymphocyte cells. Risk SNPs (rs2273596 and rs7160654) are respectively disrupting response elements for NHLH1 (Nescient helix-loop-helix) and NR3C1 (nuclear receptor subfamily 3, group C member 1). The NHLH1 transcription factor correlates with DNA methylation and gene expression in the brains of patients with bipolar disorder and schizophrenia^22^. NR3C1, also known as the glucocorticoid receptor, is involved in multiple aspects of the inflammatory response.

### Expression Quantitative Trait Loci (eQTL) associations

Using data from the Genotype-Tissue Expression consortium (GTEx), we interrogated risk SNP genotypes and gene expression in different tissues. Since the tissue overlap between REMC and GTEx was only partial, risk polymorphisms at two loci (4q21.1 and 12q12) were significantly associated (p < 2.5 × 10^−5^) with gene expression within tissues where enhancer enrichment was observed. Thus we found that the risk alleles of rs6812193 and its surrogates were associated with decreased expression of CCDC158, FAM47E, NAAA and NUP54 in many tissues including liver and fat. The risk alleles of rs76904798 and its surrogates were associated with increased expression of LRRK2 in many tissues including blood.

## Discussion

In this study we employed a novel approach to identify the potential functionality of risk-SNPs as identified by GWAS in PD. We first determined whether risk SNPs are enriched at regulatory regions in distinct cell types or tissues in the REMC database, followed by an assessment from known databases of whether and how risk SNPs disrupt response elements (transcription factor binding motifs). Although similar enrichment analyses have been employed in other studies (e.g., by Farh *et al.*^12^), this is the first time such an approach has been undertaken for PD or any neurodegenerative disease. We argue that if clusters of risk SNPs are found more often than by chance in regulatory regions (i.e., significant enrichment in that region), it is likely that alleles as defined by one or more SNPs are functional and modulate the risk of PD. In other words, alleles as defined by one or more SNP drivers carry the other SNP alleles as passengers within haplotype blocks. By this approach, we identified 12 loci significantly enriched in distinct cell types or tissues. Interestingly, only one locus with significant SNP enrichment in active regulatory regions was found in the substantia nigra. Our conclusion is that the risk SNPs identified by GWAS are likely involved in biological mechanisms conferred by cell types beyond the substantia nigra dopaminergic neurons that have traditionally been the focus of PD risk.

Despite a dramatic increase in knowledge regarding the genetics of PD during the past decade^4^,^5^,^6^, the precise underlying pathogenetic mechanisms have remained elusive. Indeed, the contributing mechanisms might differ greatly among PD patients, because the clinical features, age at onset and rate of disease progression vary significantly^1^. The emerging picture of PD is one of a clinical syndrome with different underlying etiologies and molecular mechanisms, rather than a single disease^1^,^5^,^23^. This concept is consistent with the idea that changes in multiple independent molecular pathways, and the genes that control their activities, might influence the risk for PD. Several studies have implicated an impairment of the lysosomal autophagy system; mitochondrial deficits, oxidative stress, and neuroinflammation as key upstream events in the disease process^24^. This concept also agrees with the emerging view that the initial molecular triggers for PD might not always occur in the cell types that eventually bear the brunt of the disease, i.e., the midbrain dopaminergic neurons and other neurons that show exhibit α-synuclein aggregates. The concept of such “non-cell autonomous” toxicity has gained recent support^25^ and stresses the need to also study non-neuronal cells when searching for the causes of PD.

We observed that genetic variants in lymphocytes, mesendoderm, brain, liver- and fat-cells could all be involved in PD risk, which is consistent with the theory that PD risk is affected by changes in multiple independent molecular pathways and the genes that control their activities; in different cell and tissue types, even outside the brain. Indeed, only one of the risk loci we identified has risk SNP enrichment in substantia nigra. Despite the traditional research focus on nigral dopaminergic neurons, pathological alpha-synuclein aggregates are present throughout both the peripheral nervous system and brain^26^ and they are believed to first appear in the olfactory system and in the nerves innervating the gastrointestinal tract and heart^27^, possibly as early as 5–15 years before diagnosis^28^,^29^. This phase is entitled “prodromal PD” and excludes overt motor symptoms, but it is associated with other functional deficits, e.g., anosmia, sleep disorder and constipation^28^,^29^. Considering the length of this prodromal phase, genetic variations that influence the pathogenic mechanisms, even in a minor way and outside the nervous system, could conceivably profoundly impact lifetime PD risk. Although this paper does not attempt to establish the exact mechanisms by which the identified loci confer risk, in the following sections we discuss hypothetically how they may be involved in the pathobiological mechanisms of PD.

Three of the risk loci we identified are related to the immune system. Neuroinflammation is an integral part of the disease process in PD^30^,^31^,^32^, with ample evidence available for both activated microglia and increased levels of pro-inflammatory cytokines^33^. Taken together, this brings changes in the genetic regulation of immune cells to the center stage. Two of the PD risk SNPs were enriched in CD19^+^ B-lymphocytes, at enhancers for LRRK2 (chromosome 12) and at another locus (chromosome 14) the disruption of the glucocorticoid receptor response element. The immune system might be relevant to PD in at least three fundamentally important pathogenetic aspects: (i) interactions of the immune system and α-synuclein aggregation; (ii) autoimmunity; and (iii) changes in host defenses against pathogens.

Inflammation has been suggested to be a potential trigger of Lewy pathology. Specifically, neuroinflammation promotes α-synuclein misfolding in the brain^34^, and inflammation in the gut has been proposed to cause α-synuclein aggregation in enteric nerves starting a cascade of propagating Lewy pathology via the vagal nerve^35^,^36^,^37^. As stated above, one of the risk loci in B-lymphocytes is localized at an enhancer region within *LRRK2*, a gene which can harbor mutations that collectively are the most common known genetic cause of both familial and sporadic PD. *LRRK2* is also associated with the inflammatory bowel disorder Crohn’s disease, hinting that it is involved in regulating intestinal inflammation^38^. The LRRK2 protein possesses both kinase and GTPase activities. The precise function of LRRK2 is not fully understood and expression of the protein in nigral dopamine neurons remains controversial. On the other hand, LRRK2 is expressed in immune cells including monocytes, microglia and CD19 + B-lymphocytes, and is up-regulated following recognition of microbial structures^39^. Therefore LRRK2 has been suggested to regulate immune responses in the brain, and microglia lacking LRRK2 exposed to proinflammatory stimuli have attenuated responses^40^,^41^. Brain microglia can phagocytose extracellular α-synuclein, reduce neuron-to-neuron transfer of α-synuclein and thereby potentially play a protective role by limiting the propagation of α-synuclein pathology between brain regions^42^,^43^,^44^. Changes in LRRK2 mediated signaling have been suggested to alter clearance of misfolded alpha-synuclein from the brain and these effects might at least in part be mediated by microglia. Interestingly, where comparisons could be made between the GTEx and REMC databases, two of the enriched risk loci (rs6812193 and rs76904798) were associated with near-cis (+/− 1000 kb) gene expression in the same tissues, indicating tissue-specific functionality of the risk enhancers involved. It is unclear how the eQTL genes at the two loci participate in PD risk, with the notable exception of LRRK2. Thus, the positive direction of association between risk alleles of rs76904798 and LRRK2 expression is in line with the known gain-of-function Mendelian mutations in this gene, which in turn lead to PD.

Despite that reactions in brain tissue are more tightly controlled than in the peripheral tissue, peripheral immune cells can readily access the brain^45^ and expression of risk loci in B-lymphocytes might directly contribute to inflammatory mechanisms in PD pathogenesis. Under physiological conditions, memory- and regulatory T-cells, and most likely also regulatory B-cells, traffic and monitor the central nervous system tissue and react to damage and invading pathogens^46^. Potential aberrations in these patrolling immune cells could lead to autoimmunity or unrestricted immune reactions reactions. Co-stimulatory receptors expressed on B cells include CD19, a B-cell activating factor receptor and Toll-like receptor. Hyperactivity of these receptors may break B-cell tolerance in several autoimmune diseases. B-lymphocytes could also be important in PD pathogenesis considering their key role in the primary host defense against pathogens and the evidence that B-cell-related genes, including CD19, are significantly down-regulated in peripheral blood leukocytes from patients with PD^47^,^48^. Moreover, the absolute numbers of circulating CD19 cells are reduced in PD patients^49^. Another risk locus, possibly regulating FAM47E, was present in liver and adipocytes. In the liver, processes such as oxidation and the detoxification of metabolites and exogenous agents might contribute to PD. States with increased metabolic inflammation, such as diabetes, are coupled to an increased vulnerability of dopaminergic neurons to certain neurotoxins^50^,^51^,^52^. Furthermore, changes in liver cell function could impact uric acid metabolism. This would be important because changes in uric acid have been suggested to protect against PD, via its antioxidant effects of urate, and changed liver function is associated with changes in urate levels^53^. The liver harbors a large population of monocytes and macrophages that functions as phagocytes, breaking down pathogens and toxins, and that also presents antigens to lymphocytes^54^.

Possible effects of neurodevelopmental changes cannot be overlooked. A diagnosis of PD requires motor symptoms that are consequential to severe degeneration of nigral dopaminergic neurons and their striatal terminals^55^. The number of nigral dopaminergic neurons varies greatly among normal human subjects^56^,^57^ and between different inbred strains of mice^58^, Thus, genetic factors might affect dopaminergic neurogenesis in humans and one can postulate that individuals born with more nigral dopaminergic neurons are less likely to be diagnosed with PD during their lifetime. Mesendoderm gives rise to, *inter alia*, mesodermal cells^59^, which in turn gives rise to (again *inter alia*) the notochord. The notochord releases hedgehog signaling proteins which are necessary for the development of the floor plate^60^,^61^ -an ectodermal tissue that forms neural progenitors including those that differentiate into midbrain dopaminegic neurons^62^. Our analysis of the *8p22* PD risk locus revealed disruption in several transcription factor response elements that are active in BMP4-treated ES-derived mesendoderm. It is conceivable that they affect the differentiation of the notochord; thereby they might have an effect on lifetime PD risk by reducing the number of midbrain dopaminergic neurons generated by the floor plate.

## Concluding remarks

**Figure 6:**
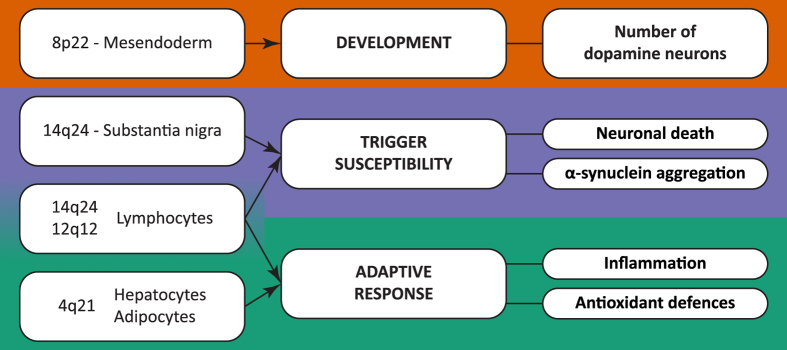
Outline of the novel connections made in this study.

Genetic studies have led to novel insights in risk mechanisms of complex diseases, especially in studies of the genetic predisposition to cancer^63^. Despite this, many identified risk loci still remain challenging to functionally assess, due to the presence of too many potentially functional SNPs and lack of knowledge of the 3D structure of chromatin, making it difficult to link enhancers with their target genes. Annotating regulatory risk regions within specific cell types, as reported here, will go a long way toward understanding the risk mechanisms of complex genetic diseases. The putative risk mechanisms for PD are summarized in Fig. 6, in which we have considered developmental, trigger, and adaptive response mechanisms. The work reported here represents a novel strategy for identifying biological mechanisms that might influence PD risk and, importantly, provides new insight into the etiology of this devastating and progressive neurological disorder. We are currently developing laboratory models to investigate the mechanisms of these genetic associations within their appropriate organ and cellular contexts as informed by our enrichment analysis.

## Methods

### Functional annotation of GWAS

Twenty-six index SNPs associated with PD were downloaded from http://www.pdgene.org/top_results. Five SNPs were removed from further analysis. Rs17649553 was removed because it was in an assembly exception. Rs11060180, rs34311866, rs356182 and rs62120679 were removed because they had no SNPs with r^2^ ≥ 0.8. This left 21 SNPs for further enrichment analysis. These SNPs were integrated with comprehensive data from the Roadmap Epigenomics Mapping Consortium (REMC) for 77 tissues and cell types and 1000 genomes using FunciSNP software^11^. For each of the cells and tissues from REMC, consortium IDR-processed data^64^ were obtained for each of three data types, H3K4me1 and 3, and H3K27Ac. SNPs in LD at r^2^ ≥ 0.8 and overlapping these features were identified using FunciSNP software. In order to functionally categorize the SNPs, we used a rules-based segmentation algorithm to classify regions in every listed cell type based on histone marks. The following describes the rules in order of decreasing precedence. Regions with a positive signal for the histone modification H3K4me3 were classified as promoters, and as active or poised on the basis of overlap with the active histone mark H3K27ac (promoter active region PAR). H3K4me1 regions were classified as enhancers, and similarly as active or poised on the basis of overlap with H3K27ac (enhancer active region EAR). Each region had a single annotation assigned such that SNPs could be assigned one of three unambiguous functions based on the combinations of histone marks. The 77 REMC cell types used for the study and numerical significance of enrichment (see below) are listed in Supplemental Table 1. Cell type abbreviations are listed in Supplemental Table 2. The enrichment is also presented as a heatmap in Fig. 1 for visual simplicity.

### Enrichment analyses

We used the hypergeometric distribution to calculate the probability of the overlaps against the background of 1000 genomes (within 1 MB windows around the index SNPs inclusive) and adjusted for multiple comparisons for each cell type with FDR < 0.05. This was repeated for the pooled categories EAR & PAR (active enhancers and promoters). Enriched regions were evaluated in the UCSC genome browser with wiggle tracks (.wig) for H3K4me1 and H3K27ac, our genome segmentations and tissue-specific FunciSNP results. We calculated a Euclidean distance metric for log-scaled p values for each index SNP in each tissue and used average linkage hierarchical clustering with the heatmap. 2 package in R (Fig. 1).

### Analysis of Transcription Factor binding sites

SNPs located within enhancers or promoters of any kind were analyzed using the bioconductor motifbreakR package using default method settings (weighted sum) with a p-value cutoff at 5 × 10^–5^ (ref. 10). Figures were prepared from screenshots of the UCSC genome browser and MotifbreakR plotting functions for SNPs at p < 10^–5^.

### Expression Quantitative Trait Loci (eQTL)

All polymorphisms within +/− 50 kb of the FAM47E or LRRK2 locus which were included the meta-analysis by Nalls *et al.*^7^ and associated with a p-value of at least 0.05 were downloaded from pdgene.org. To these were added unique SNPs in LD (r^2^ ≥ 0.8) found via the Broad Institute SNAP program (https://www.broadinstitute.org/mpg/snap/). This enlarged set was used to subset the significant eQTL SNP-gene associations available from the GTEx consortium (http://www.gtexportal.org/home/). This subsetting produced a tissue specific collection of SNP-gene expression associations linked to PD.

### Computers and Software

Genome segmentations, FunciSNP, clustering, motifbreakR, and SNP-tissue enrichment analyses were carried out in R (https://cran.r-project.org) on an iMac Retina 5k with a 3.5-GHz processor and 24 GB of memory.

## Additional Information

**How to cite this article**: Coetzee, S. G. *et al.* Enrichment of risk SNPs in regulatory regions implicate diverse tissues in Parkinson’s disease etiology. *Sci. Rep.* **6**, 30509; doi: 10.1038/srep30509 (2016).

## Supplementary Material

Supplementary Information

Supplementary Table 1

Supplementary Table 2
