# Supplementary material for: Enrichment of risk SNPs in regulatory regions implicate diverse tissues in Parkinson’s disease etiology
Source: Sci Rep. 2016 Jul 27;6:30509. doi: 10.1038/srep30509 (PMC4962314; doi:10.1038/srep30509)

Enrichment of risk SNPs in regulatory regions implicate diverse tissues in Parkinson's disease etiology

Simon G Coetzee[1], Steven Pierce[2] Patrik Brundin[2], Lena Brundin[2], Dennis J Hazelett*[1] Gerhard A Coetzee*[2]

[1]Bioinformatics and Computational Biology Research Center, Biomedical Sciences, Cedars-Sinai Medical Center, Los Angeles, California, USA

[2]Center for Neurodegenerative Science, Van Andel Research Institute, Grand Rapids, Michigan, USA

Legends

Supplemental Table 1:

Negative natural (Ln) log p-values in enrichment of correlated SNPs (r^2>0.8) at 21 GWAS hits (columns) in 77 REMC cell types (rows). Color code relates to ranked multiple hypotheses-corrected and locus-specific negative LN p-values. The black bordered cells were chosen for further in-depth analysis (Figs 2-6). Data are also presented as a heat-map in Fig 1 for easy visualization ($-\log_e P > 3$ corresponds to multiple tested corrected $p < 0.05$). The loci selected for further analysis had highly significant values ranging from 14.3 - 23.0.

Supplemental Table 2:

REMC Cell type name Abbreviations

Supplemental Fig 1:

Schematic workflow employed in this study.

Schematic Workflow

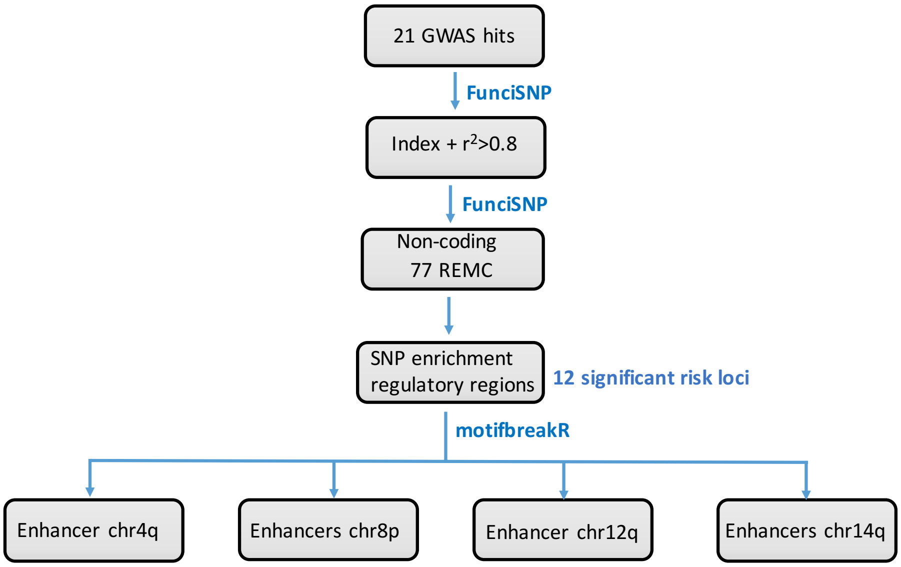

Supplement: Supplementary Information [file srep30509-s1.pdf]
